# An Experimental and Simulation Study on the Formability of Commercial Pure Titanium Foil

**DOI:** 10.3390/mi15091096

**Published:** 2024-08-29

**Authors:** Jenn-Terng Gau, Kechuang Zhang, Jiaqi Zhu

**Affiliations:** Department of Mechanical Engineering, Northern Illinois University, DeKalb, IL 60115, USA; z1720609@students.niu.edu (K.Z.); z1804486@students.niu.edu (J.Z.)

**Keywords:** formability, microforming, commercial pure titanium grade 2, micro LDH test, FLC

## Abstract

In order to understand the formability of as-received tempered commercial pure titanium grade 2 foils (CP Ti Gr2) with a thickness of 38 µm, a series of micro limited dome height (µ-LDH) tests were conducted in quasi-static speed (0.01 mm/s) at room temperature without the use of a lubricant. A technique developed at NIU was also used to create micro-circular grids (*ϕ*50 μm) on the as-received material. The forming limit curve (FLC) of the CP Ti Gr2 foils was obtained through the proposed µ-LDH test. For having mechanical properties of the CP Ti Gr2 foils for LS-Dyna FEA (Finite Element Analysis) simulations, a series of tensile tests in three directions were also conducted at room temperature with the same speed. The obtained FLC has been validated using a micro deep drawing case study in which both FEA simulations and experiments were conducted and compared. It has been proven in this study that the FLC obtained using the proposed µ-LDH test can be used for an extremely thin sheet-metal-forming process by the automotive, aerospace, medical, energy, and electronic industries, etc., right away for product design, forming process development, tool and die designs, and simulations, etc.

## 1. Introduction

Commercial pure titanium (CP Ti) has been widely utilized in industries due to its lightweight, high specific strength, excellent corrosion resistance, and biocompatibility, etc. CP Ti has received much more attention from the medical and electronic industries for manufacturing micro components with a greater long-term reliability. Compared to the main current biomedical materials such as stainless steel, cobalt alloys, and titanium alloys, CP Ti is considered to be the best biocompatible metallic material that allows for bone tissue growth to adhere to implants for a longer time than those made of other biomedical materials. It is widely used to fabricate implant devices such as artificial hip joints, micro screws for fracture fixation, dental implants, etc. [1]. According to the information from Arnold Magnetic Technologies [2], the CP Ti foils have also been used to produce high-end speaker domes in electronics, fuel cell bipolar plates, and solar panel lamination for the energy industry, etc. For example, Jin et al. [3] conducted an experimental study to form titanium bipolar plates for proton exchange membrane fuel cells by using a rubber forming process. The thickness of the titanium in their study was 100 µm and their experiments were conducted with a 200-ton hydraulic press. The need of micro thin-wall CP Ti parts (thickness ≤ 50 µm) has been tremendously increased due to the trend in miniaturization in the medical, aerospace, sensor, electronic, automotive, and energy industries, etc. However, among the micro manufacturing processes, the micro foil forming process is the most cost- and time-effective process to produce thin-wall parts with extremely tight tolerance in mass production. However, CP Ti exhibits low formability and strong plastic anisotropy because of its HCP crystal structure. Therefore, understanding the formability of the CP Ti foils becomes very essential for product design, process development, die design, and FEA simulations, etc. This is the reason why the formability of the CP Ti Gr2 foils has been studied in this study.

Forming limit diagram (FLD) is a graphic description for characterizing the formability of sheet metal. After the concepts of FLD were first introduced by Keeler [4] and Goodwin [5]. Many efforts have been made to develop theoretical methods to predict FLCs for various materials in different manufacturing process. Swift’s diffuse instability criterion [6] was the first analytical approach to predict FLC for stretching deformation. Another approach is Hill’s localized instability criterion [7], which predicts deep drawing deformation in a negative minor strain region. The M-K theory predicts the FLC based on the assumption of an initial defect in perpendicular direction with respect to loading direction. In addition, some analytical models for predicting FLC are based on ductile fracture criteria [8]. Because there are so many different theoretical models to predict FLC, experimental methods are becoming more acceptable for industries to determine FLC and at the same time to verify these analytical models.

As is well known in the sheet metal forming industry, FLDs are influenced by microstructure, material properties (e.g., strain hardening exponent, anisotropy coefficients, and strain rate sensitivity), and process parameters (e.g., binding force, frictional coefficient, temperature, strain rate [9]). For investigating the formability of CP Ti sheets, many formability studies were conducted in a macro-scale sheet metal forming process. For instance, Stachowicz et al. [10] studied the microstructure effect on the limit strains of CP Ti sheets annealed at different temperatures by carrying out bulge tests. The limit strain of the CP Ti sheet increases with an increase in grain size. Adamus [11] showed that decreasing binding force and friction during the deep-drawing process would improve the material flow that results in a more balanced strain distribution and thus enhances the drawability. Chen and Chiu [12] examined the thermal effects on the formability of commercial pure titanium sheets with a thickness of 0.5 mm using a series of tensile tests, v-bend tests, circular cup drawing tests, and dome height tests conducted at different temperatures. It has been observed in their study that the flow stress curve becomes lower proportionally with an increase in the testing temperature, and the formability can be improved by increasing the forming temperature. However, the knowledge and techniques developed at the macro scale cannot be directly applied to the micro foil-forming process due to the so-called size effects. Vollertsen [13] characterized the size effects into three main categories: density, shape, and microstructure effects. Gau et al. [14] conducted a series of experiments to understand the influence of size effects on the formabilities of aluminum 1100 and brass. In addition, Gau et al. [15] also investigated the coupling influence of size effects and strain rates on the formability of austenitic stainless steel 304 foil through a series of micro-scale limited dome height (µ-LDH) tests conducted in three different punch speeds without applying lubricant on the annealed and as-received foils.

It is essential to have the FLC of the CP Ti foils for forming process development, simulation and die designs in order to form the CP Ti extremely thin wall products. In this study, *ϕ*50 µm micro circular micro grids were created on the as-received CP Ti Gr2 foils (38 µm) before experiments. The diameter of the hemisphere punch used for the micro LDH test and punch speed were 2 mm and 0.01 mm/s, respectively. The results of this study would enhance our abilities to form micro scale parts or extremely thin 3D parts with micro features such as micro channels on the CP Ti Gr2 foils. The potential applications of the results of this paper include, but are not limited to, fuel cell bipolar plates, sensors, electronics, and micro fluidic and biomedical devices, etc. For the purpose of validation, the obtained FLC has been implemented into LS-Dyna for simulations. In this study, a series of simulations were conducted and compared with the experimental result for not only virtually determining the coefficient of friction of the set of micro tool and die, but also for validating the obtained FLC.

## 2. Experimental Specimens and Experimental Setup

**Figure 1:**
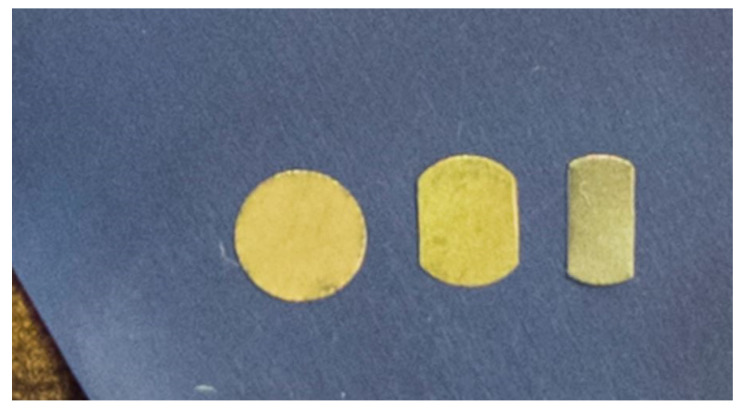
The CP Ti Gr2 foil blanks with the micro circular grids.

**Figure 2:**
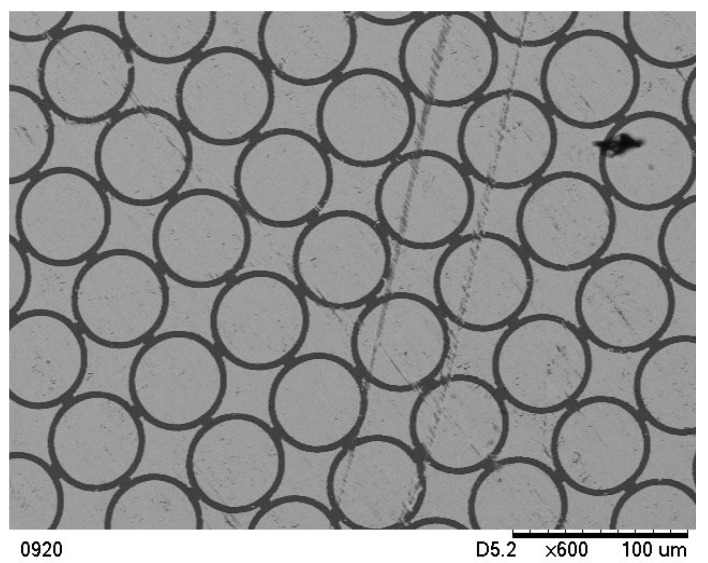
A SEM picture of the micro circular girds (*ϕ*50 µm) on a specimen [16].

The as-received CP Ti Gr2 foil with a thickness of 38 µm from Arnold Magnetic Technologies in Illinois was used for this study. The mechanical properties, chemical composition, and physical properties can be found in Table 1, as provided by the manufacturer. The data in Table 1 can be used for reference only because, normally, material suppliers only need to provide the lower bound data according to industry practices and standards. Therefore, it is necessary to conduct tensile tests in order to have the precise mechanical properties of the as-received materials. The as-received foils were blanked into 50.8 mm × 50.8 mm square blanks first, and then the pure gold (Au) micro circular grids (*ϕ*50 µm) were created on the blanks (one side only) before being blanked into the specific shapes for experiments. Figure 1 shows the µ-LDH test specimens (4 mm, 3 mm, and 2 mm) with micro circular grids, while Figure 2 is a SEM picture taken using a Hitachi TM-1000 Tabletop SEM (Scanservice Corporation, Los Angeles, USA) with 600 times magnification, showing some micro-circular grids on a specimen before deformation. Because the created pure gold (Au) micro-circular grids are extremely thin (≈50 nm) with a much higher ductility in comparison with the CP Ti Gr2, and because the base material has not been etched at all, the experimental results are very accurate.

**Figure 3:**
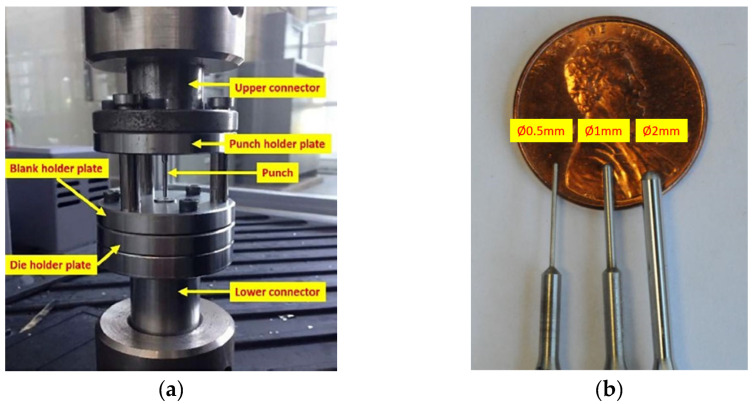
(**a**) The setup for the µLDH test in MTS 43 [17]. (**b**) Punches used for the µLDH test.

**Figure 4:**
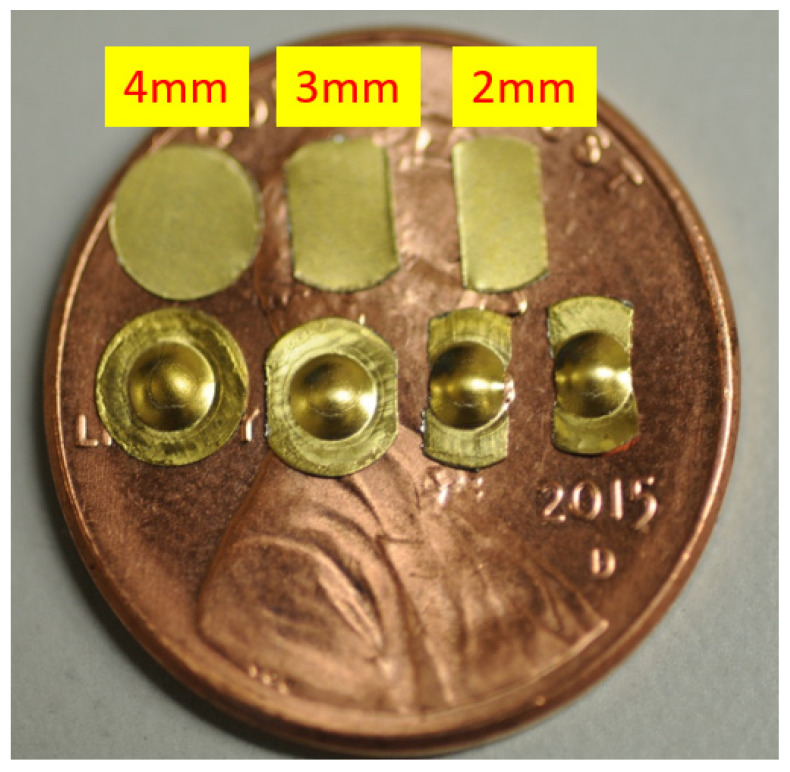
Micro LDH test specimens (before and after testing) on a US penny.

In this study, a series of µ-LDH tests were conducted using a MTS 43 machine as the testbed with 0.01mm/s punch travel speed. The setup for this test on MTS 43 is shown in Figure 3a, while Figure 3b shows the three hemisphere punches (*ϕ*2 mm, *ϕ*1 mm, and *ϕ*0.5 mm) used at NIU, but only the 2 mm punch was used for this study. For obtaining different deformation modes (biaxial tension, stretching deformation, plane strain, and deep-drawing modes), three different specimen sizes and geometries (4 mm, 3 mm, and 2 mm) were designed and tested. The 4 mm specimens were used to obtain biaxial tension mode, while the 3 mm specimens were used for stretching the deformation mode. Both the plane strain and deep-drawing modes can be obtained by testing the 2 mm specimens through adjusting the boundary conditions on the binders. The purpose of having different deformation modes (biaxial tension, stretching deformation, plane strain, and deep-drawing modes) is for constructing the FLD with an FLC. Figure 4 shows the specimens before and after the experiments. In the picture, two deformed parts right below the 2 mm specimen were in plane-strain mode and deep-drawing mode, respectively.

## 3. Constructing the FLC

**Figure 5:**
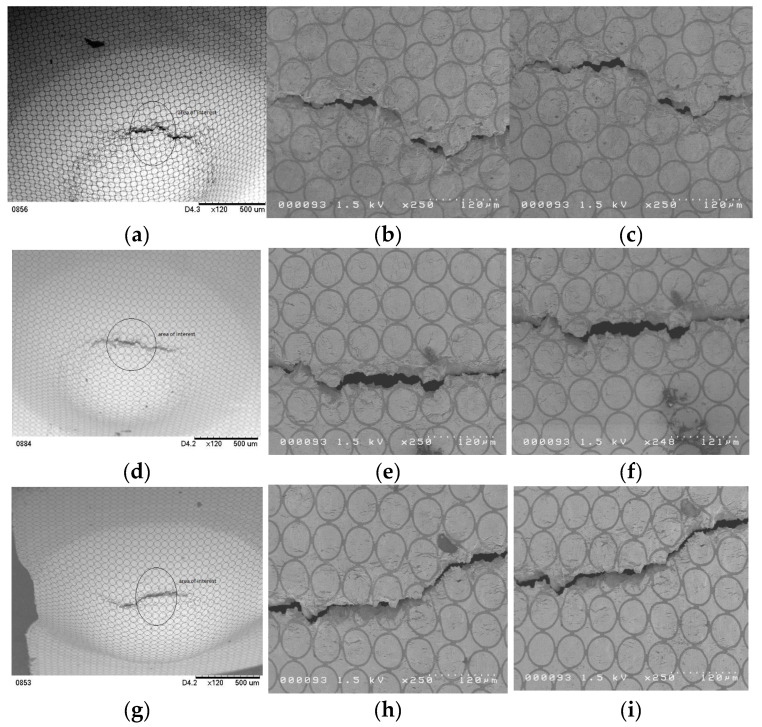

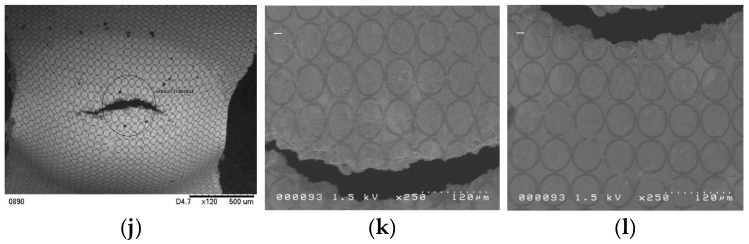
Figures (**a**–**c**) are the SEM pictures of the whole, upper, and lower parts of the fracture area of a 4 mm specimen (biaxial tension mode), while (**d**–**f**) are of a 3 mm specimen (stretching deformation mode), (**g**–**i**) are of a 2 mm specimen (plane strain mode), and (**j**–**l**) are of a 2 mm specimen (deep-drawing mode).

**Figure 6:**
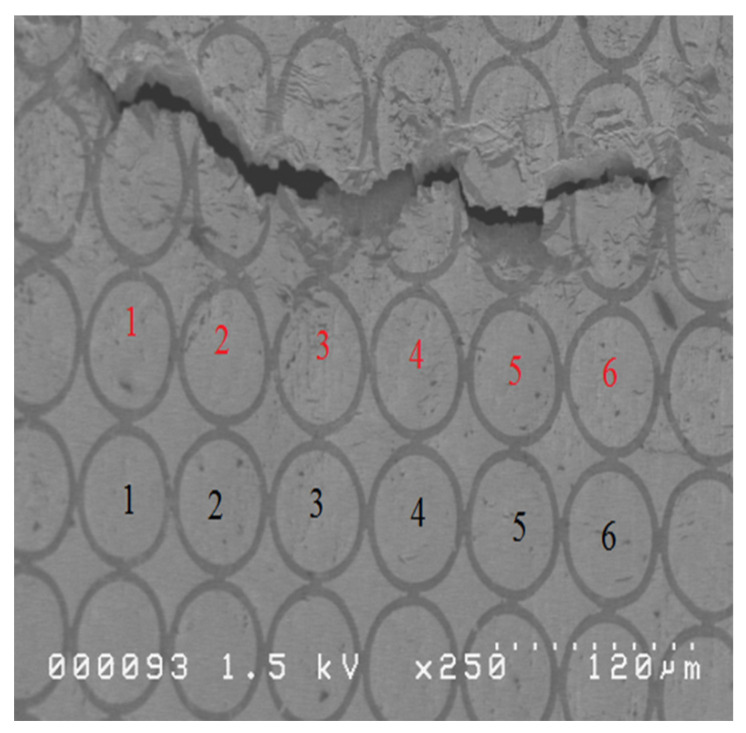
A SEM picture with the necking grids (red numbers) and the safe (black numbers) of a specimen.

**Figure 7:**
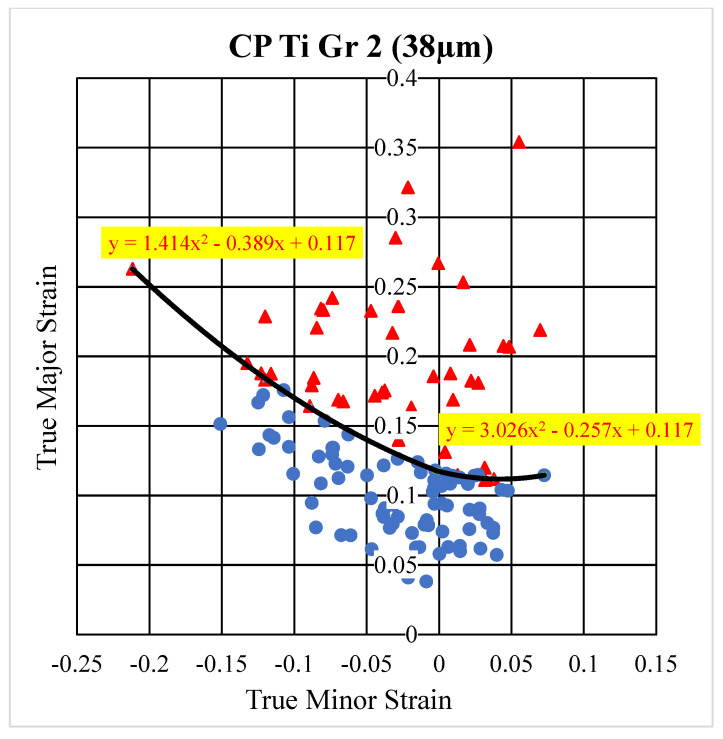
The FLD of the as-received tempered CP Ti Gr 2 foil (red triangles: necking grids; blue dots: safe grids).

At least ten specimens were tested for each deformation mode to construct the FLD. Figure 5 shows some SEM pictures of four specimens with different deformation modes; for example, pictures (a), (b), and (c) are the pictures of the whole, upper, and lower portions of the fracture area of a 4 mm specimen, respectively. The black dot in picture (a) is a dust particle in the lab. ASTM E2218-02 standard [18] was used to determine the necking grids and safe grids for constructing the FLC, and ImageJ, developed by NIH, was used to measure and compute the major and minor true strains of the selected micro grids on the SEM pictures. Figure 6 is a SEM picture with 250 time magnification of a specimen and also illustrates how to determine necking grids and safe grids from a SEM picture. The grids (with red numbers) on the row right next to the fracture area are determined to be the necking grids, while the rest are safe grids. However, it is more effective and efficient to select larger safe girds (with black numbers here) for constructing the FLC. The technique of constructing an FLC for foils can be found in a publication of Gau et al. [15]. Figure 7 shows the constructed FLD of the as-received CP Ti Gr2 foil. The red triangles and blue dots represent the selected necking and safe grids, respectively, while the two quadratic equations present the FLC for the positive and negative true strains, respectively. The quadratic equation ($y=1.414x^{2}-0.389x+0.117)$ on the left-hand side can be used for the minor true strains ranging from −0.211 to 0, [−0.211, 0], while the other one ($y=3.026x^{2}-0.257x+0.117)$ ranges from 0 to 0.072, [0, 0.072]. It has to be noted that the lowest point of the obtained FLC is not at the plane strain condition.

**Figure 8:**
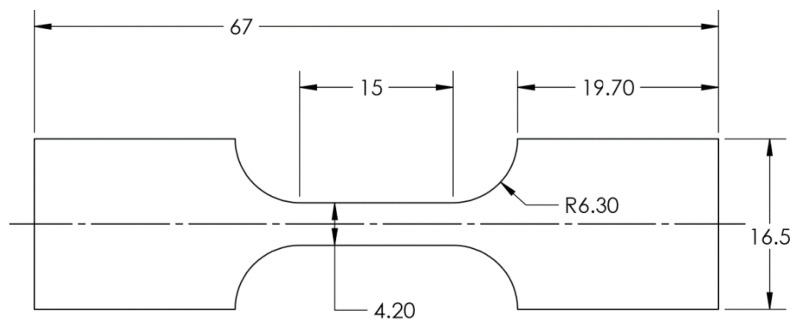
The dimensions of the tensile test specimen (unit: mm) [17].

**Figure 9:**
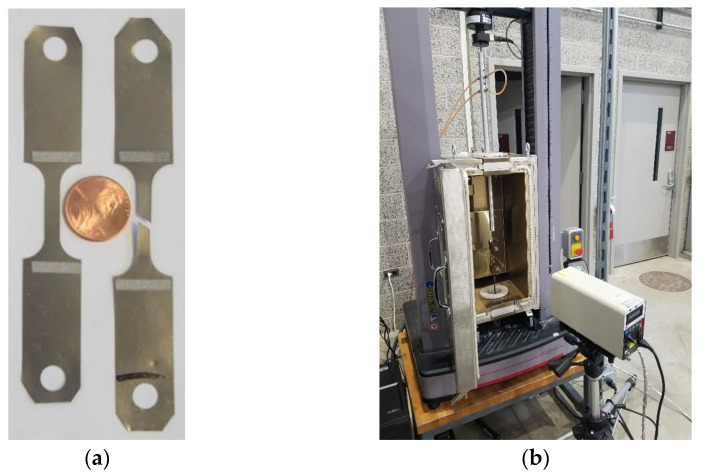
(**a**) A picture of two tensile test specimens with a US penny. (**b**) The setup for the tensile test.

A series of tensile tests were conducted at room temperature, and the geometry and dimension of the tensile test specimen is shown on Figure 8. The test speed was also set as 0.01 mm/s (0.00067s^−1^ strain rate). Figure 9a is a picture of two tensile test specimens (before and after the test), and Figure 9b displays the setup of the tensile test with an MTS laser extensometer. The results can be found in Table 2, in which S_y_, S_ut_, K, n, and ε_f_ represent the 0.2% offset yield true strength, ultimate true tensile strength, coefficient of strength, hardening exponent, and failure true strain, respectively.

## 4. Validating the FLC through a Case Study

**Figure 10:**
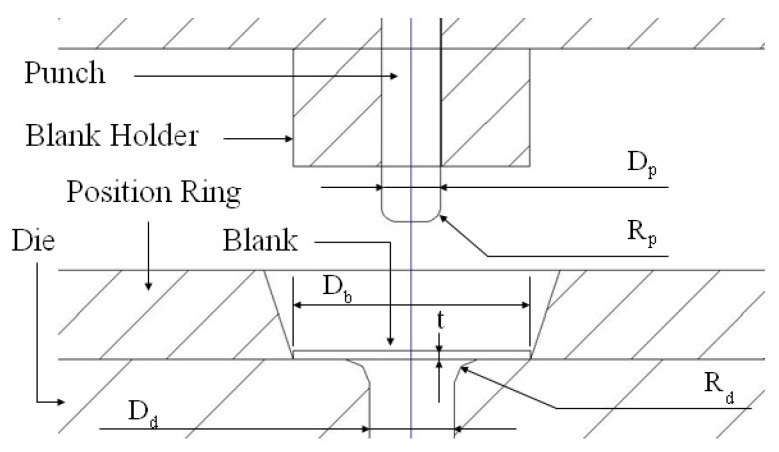
A sketch of the tooling drawing [19].

For validating the obtained FLC, a series of LS-Dyna FEA simulations and experiments were conducted for a case study. Figure 10 is a sketch of the experimental tooling for this case study. The dimensions for D_p_, R_p_, D_d_, and R_d_ were 2 mm, 0.25 mm, 2.22 mm, and 0.4 mm, respectively. In this case study, the blank holder was removed, i.e., it had no upper binder. The reason to have this type of case study for validating the obtained FLC is explained as follows.

In the micro deep-drawing process, the micro cup is cracked due to extreme thinning happening on the draw wall. There are two possibilities causing the exceeding thinning: (1) the blank holder force is too large, so the material cannot be sufficiently drawn into the die cavity; (2) when the cup has the severed wrinkles, the material is stuck between the punch and die, and then no material can be drawn into the die’s cavity. However, regardless of the reason, the same FLC can be used when the material remains the same. According to the experience of the authors, drawing a micro cup without the binder present (possibility 2) is more complicated than with a binder. Through this case study, not only the coefficient of friction can be virtually determined, but also the FLC, initial fracture location, and cup geometry (with sever wrinkles and shape) can be validated and evaluated.

**Figure 11:**
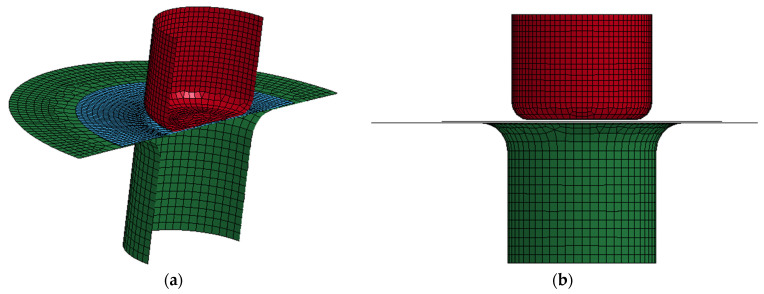
LS-Dyna FEA model for simulation: (**a**) section view and (**b**) front view.

**Figure 12:**
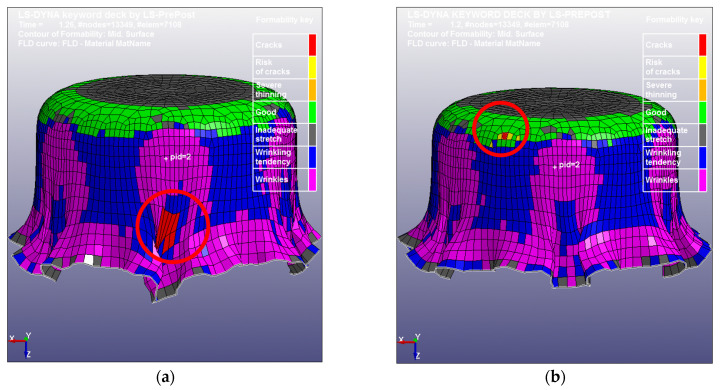

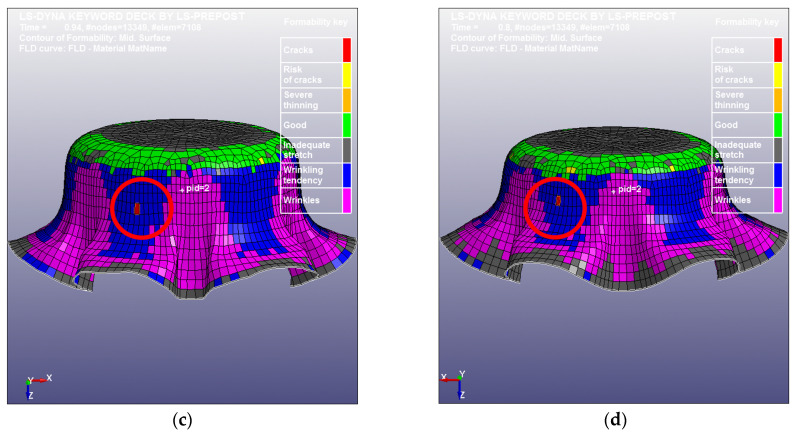
The simulation results: (**a**) μ = 0, (**b**) μ = 0.1, (**c**) μ = 0.2, and (**d**) μ = 0.3.

The diameter and thickness of the blanks used in the simulation were 4 mm and 38 μm, respectively, e.g., the 4 mm blank shown in Figure 4. Table 3 lists the mechanical properties of the as-received CP Ti Gr2 for the simulation. Figure 11 shows the LS-Dyna FEA mesh model for these case study simulations. The 037-Transversley Anisotropic Elastic Plastic NLP Failure was used as the material model, and the punch and the die were defined as rigid bodies by using the 020-Rigid material model. The adaptive mesh function was turned on for the blank, and the punch travel speed was set as 1 mm/ms for minimizing the CPU time. Four different coefficients of friction (0, 0.1, 0.2, and 0.3) were used for four different simulations because the coefficient of friction was unknown; no lubricant was used in the experimental part of this case study. Through the aid of simulations and the comparisons with the experimental drawn cup, the coefficient of friction could be virtually determined and validated. Figure 12 shows the results of the four simulations with four different coefficients of friction (0, 0.1, 0.2, and 0.3). In Figure 12, the initial fracture locations (the elements with red color indicating those with cracks) are highlighted by red circles. The obtained FLC has been imported into LS-Dyna to determine whether the elements are cracked or not. By comparing the results in Figure 12, it is obvious that the micro cup can be drawn deeper with a decreasing coefficient of friction. In addition, the friction also has effects on the geometries and sizes of the wrinkles of the cup when no binder was used

**Figure 13:**
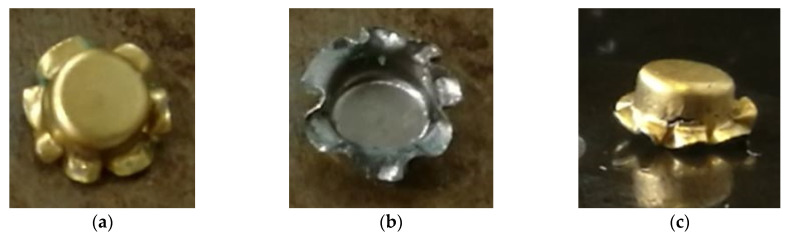
The drawn cup in this study: (**a**) top, (**b**) bottom, and (**c**) side views.

**Figure 14:**
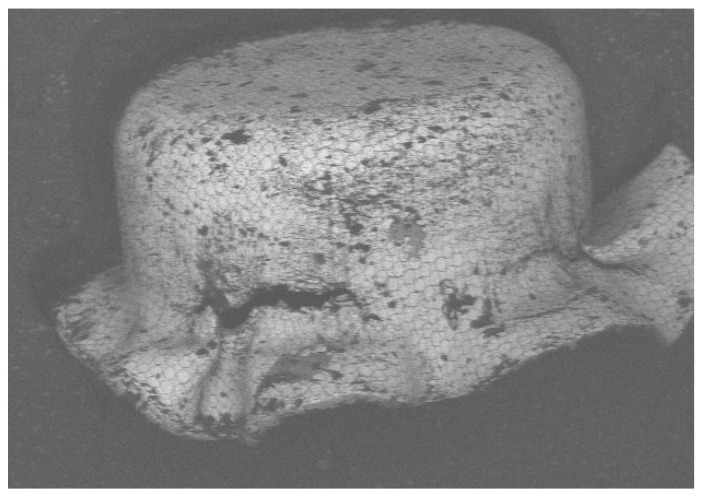
A SEM picture of the drawn cup for the case study.

**Figure 15:**
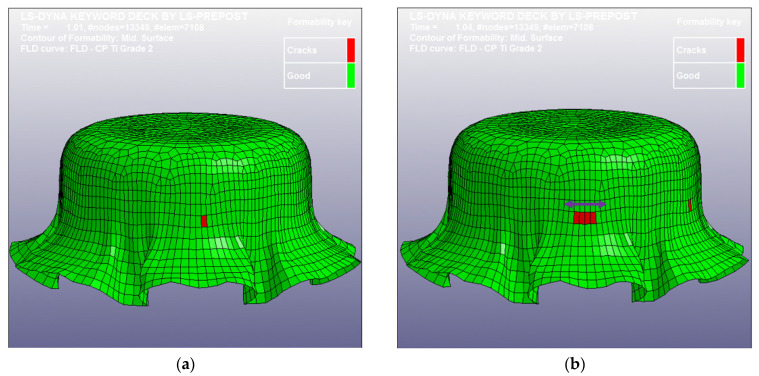
The simulation results with µ = 0.15: (**a**) initial crack and (**b**) crack propagation.

**Figure 16:**
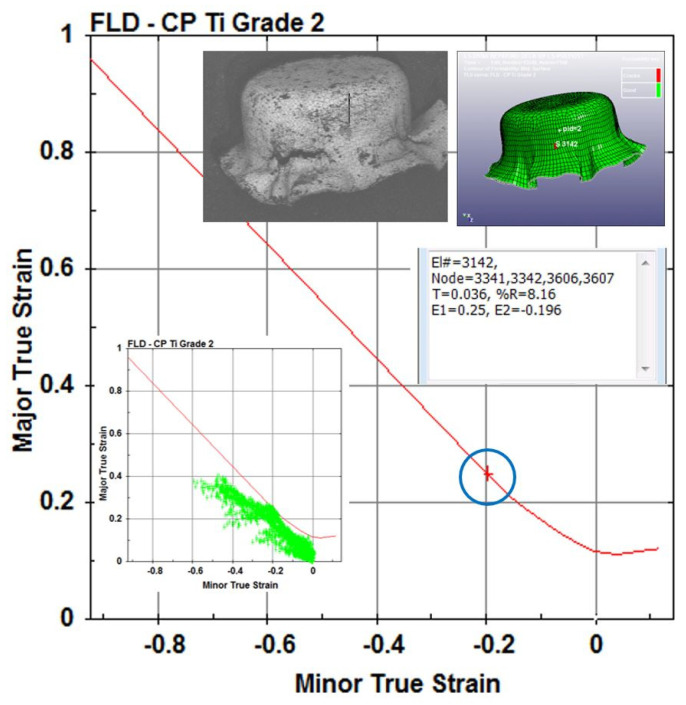
A comparison of the simulation and experimental results.

Figure 13 shows three pictures (top, bottom, and side views) of the experimental cup drawn by the tooling shown in Figure 10 without a binder. By comparing the simulation results with the physical drawn cup, the simulation with µ = 0.2 is the case most closed to the drawn cups in this study. Therefore, the most reasonable coefficient of friction should be very close to 0.2. Figure 14 displays a SEM picture of the drawn cup, referring to the picture (c) in Figure 13. Some *ϕ*50 µm micro circular grids on the cup surface can also be observed clearly from the SEM picture. According to the experimental data, the cup started cracking when the punch travel distance was about 1.0 mm. Therefore, many iterative simulations through tuning the coefficient of friction were run. It was found that the punch travel distance of the simulation with µ = 0.15 was 1.01 mm when the cup just had the initial crack. Figure 15 presents two pictures of the simulation with µ = 0.15, while Figure 16 shows a comparison of the experimental and simulation results. As shown in Figure 16, the shape of the cup, punch travel distance, and location of the initial crack obtained from the simulation with µ = 0.15 are almost identical with the drawn cup. Therefore, the coefficient of frication has been virtually determined to be 0.15. In addition, the initial crack element (# 3142) was detected using the FLC obtained in this simulation. The major and minor true strains of the initial crack element (# 3142) are 0.25 and −0.196, respectively, and it is located (highlighted by a blue circle) right above the FLC. Therefore, it has been proven by this case study that the obtained FLC is correct and can be used right away for FEA simulations, product designs, forming process development, and die designs, etc.

## 5. Conclusions

A series of µ-LDH tests without lubricant were conducted on the as-received CP Ti Gr 2 foils (38 µm) at room temperature with 0.01mm/s punch travel speed for obtaining its FLC. Tensile tests were also conducted to have the mechanical properties in the rolling, diagonal, and transverse directions. In addition, an experimental and numerical simulation case study on the micro deep-drawing process (without a binder) was also conducted for validating the obtained FLC. The coefficient of friction was unknown during experiments, i.e., µ-LDH tests and micro-deep drawing studies; however, it has been determined through a series of iterative simulations by tuning the coefficient of friction. By comparing the simulations with the physical drawn cup, the coefficient of friction for this set of tooling and processes was determined. This technique and process of virtually determining the coefficient of friction can not only be used for the micro foil forming process, but also other types of microforming processes, such as micro extrusion.

Some conclusions from this study are summarized below:

- The proposed µ-LDH tests can be used to create FLCs of the extremely thin sheet metals (foils).
- The FLC of the as-received CP Ti Gr 2 foils was created.
- The FLC has been implemented into LS-Dyna for micro forming simulations and validated using a micro deep drawing case study.
- The coefficient of friction can be virtually determined through a series of simulations and comparisons with physical parts.
- The obtained FLC can be used for product design, forming process development, and tool and die design, etc., for micro extremely thin-sheet forming.
- The micro cup can be drawn deeper with decreasing the coefficient of friction. It means that friction has a negative impact on the formability.

## Figures and Tables

**Table 1:** The properties of the CP Ti grade 2 foils (data from Arnold Magnetic Technologies [2]).

Mechanical Properties		Chemical Elements (%)		Physical Properties	
E	116 GPa	N	0.03	Density	4.48 g/cm^3^
G	44 GPa	C	0.08	Beta Transus	915 °C
ν	0.32	H	0.015	Melting Point	1668 °C
S_ut_	485 MPa	Fe	0.30	Thermal conductivity	2.179 Wm^−1^k^−1^
S_y_	345 MPa	O	0.25	Magnetic Permeability	Nonmagnetic
		Ti	99.325	Electrical Resistivity	0.53 µΩ × m

**Table 2:** The mechanical properties of the CP Ti grade 2 foils obtained from tensile tests.

**Rolling Direction (0°)**					
**Sample**	**S_y_ (MPa)**	**S_ut_ (MPa)**	**K (MPa)**	**n**	**ε_f_**
1	366	557	758.578	0.167	0.208
2	366	560	767.715	0.170	0.230
3	363	558	745.590	0.168	0.217
**Average**	$365\;(\begin{array}{c} +1\\ -2 \end{array})$	$558\;(\begin{array}{c} +2\\ -1 \end{array})$	$757.294\;(\begin{array}{c} +10.421\\ -11.704 \end{array})$	$0.168\;(\begin{array}{c} +0.002\\ -0.002 \end{array})$	$0.218\;(\begin{array}{c} +0.012\\ -0.010 \end{array})$
**Diagonal Direction (45°)**					
**Sample**	**S_y_ (MPa)**	**S_ut_ (MPa)**	**K (MPa)**	**n**	**ε_f_**
1	381	530	712.689	0.149	0.232
2	393	543	722.936	0.144	0.244
3	381	524	707.783	0.146	0.240
**Average**	$385\;(\begin{array}{c} +8\\ -4 \end{array})$	$532\;(\begin{array}{c} +11\\ -8 \end{array})$	$714.469\;(\begin{array}{c} +8.476\\ -6.686 \end{array})$	$0.146\;(\begin{array}{c} +0.003\\ -0.002 \end{array})$	$0.239\;(\begin{array}{c} +0.005\\ -0.007 \end{array})$
**Transverse Direction (90°)**					
**Sample**	**S_y_ (MPa)**	**S_ut_ (MPa)**	**K (MPa)**	**n**	**ε_f_**
1	401	531	710.722	0.137	0.232
2	396	523	703.558	0.135	0.241
3	413	548	712.853	0.131	0.227
**Average**	$403\;(\begin{array}{c} +10\\ -7 \end{array})$	$534\;(\begin{array}{c} +14\\ -11 \end{array})$	$709.044\;(\begin{array}{c} +3.809\\ -5.486 \end{array})$	$0.134\;(\begin{array}{c} +0.003\\ -0.003 \end{array})$	$0.233\;(\begin{array}{c} +0.008\\ -0.006 \end{array})$

**Table 3:** Mechanical properties of the CP Ti Grade 2 for the FEA simulation.

	Density	Young’s Modulus	Poison’s Ratio	Yield Strength	Plastic Hardening Modulus	R-Value
CP Ti Grade 2	4.51 × 10^−6^ kg/mm^3^	116 GPa	0.37	0.365 GPa	0.168	2.2

## Data Availability

Data are included in this paper.
